# Doctor as criminal: reporting of patient deaths to the police and criminal prosecution of healthcare providers in Japan

**DOI:** 10.1186/1472-6963-10-53

**Published:** 2010-02-26

**Authors:** L Jay Starkey, Shoichi Maeda

**Affiliations:** 1 School of Medicine, University of California, San Francisco, USA; 2 Graduate School of Health Science, Keio University, Tokyo, Japan

## Abstract

**Background:**

In Japan, medical error leading to patient death is often handled through the criminal rather than civil justice system. However, the number of cases handled through the criminal system and how this has changed in recent years has not previously been described. Our aim was to determine the trend in reports of patient death to the police and the trend in the resulting prosecution of healthcare providers for medical error leading to patient death from 1998 to 2008.

**Methods:**

We collected data regarding the number of police reports of patient death made by physicians, next-of-kin, and other sources between 1998 and 2008. We also collected data regarding the number of resulting criminal prosecutions of healthcare providers between 1998 and 2008. Reporting and prosecution trends were analyzed using annual linear regression models.

**Results:**

Reports: The number physician reports of patient deaths to the police increased significantly during the study period (slope 18.68, R^2 ^= 0.78, P < 0.001) while reports made by next-of-kin and others did not. Mean annual reporting rates by group were physicians 130.1 (± 70.1), next-of-kin 29.3 (± 12.5), and others 10.4 (± 6.0). Prosecutions: The number of resulting criminal prosecutions increased significantly during the study period (slope 9.21, R^2 ^= 0.83, P < 0.001). The mean annual prosecution rate was 61.0 (± 33.6).

**Conclusions:**

The reporting of patient deaths to the police by physicians increased significantly from 1998 to 2008 while those made by next-of-kin and others did not. The resulting criminal prosecutions of healthcare providers increased significantly during the same time period. The reasons for these increases are unclear and should be the focus of future research.

## Background

Medical malpractice continues to be a topic of importance around the world. As elsewhere, malpractice claims continue to rise in Japan [1]. However, Japan is unique in that malpractice cases have been increasingly handled through the criminal justice system. Doctors are being prosecuted when medical error leads to patient death [2]. In the United States, research and organizations such as the National Institute of Medicine put medical error on the political and social agenda. In Japan it was the mass media - through coverage of a case of medical error.

The case in question occurred in 1999 at Hiroo General Hospital, a well-respected government hospital in Tokyo. A nurse administered an intravenous injection of an antiseptic (chlorhexidine) after mistaking it for heparin sodium. Another nurse had left it on the cart. The patient died immediately. The case received national media attention, prompting police involvement.

The Hiroo case and subsequent cases of medical error have been handled through the usual Japanese criminal legal process, as briefly outlined here. After receiving report of a crime, police open an investigation. They then gather evidence by search, seizure, and inspection and interrogate suspects and witnesses. Police send cases they deem to have merit to the Public Prosecutors Office where prosecutors further develop the case. Note that neither the police investigators nor the public prosecutors are required to have a medical background. Cases that are sent to trial are handled either through Summary Courts for lesser offenses or through District Courts for greater offenses. Through the study period, Japan did not utilize trial by jury, though some criminal cases will be tried by jury from early 2009. In the District Courts, one to three judges preside, depending on the severity of the crime. The court hears the cases of the public prosecutor and the defense. After deliberation, the court pronounces a formal verdict, its rationale, and the punishment. Punishment may include fines, administrative action including license suspension or revocation, and imprisonment.

Through the above process, the healthcare providers involved in the Hiroo case, including two nurses, an attending physician, and the hospital director, were prosecuted, found guilty, and sentenced with fines and commuted prison terms. The Supreme Court upheld the ruling on appeal.

During the trial, a previously obscure law called Article 21 of the Medical Practitioner's Law became the crux of the prosecution's case. Article 21 requires physicians to report any "unusual death" occurring under "suspicious circumstances" to the police within 24 hours. Originally, the provision was intended for cases of foul play. However, in 1994 an influential legal society interpretation extended the reporting duty to include cases where medical treatment could have caused or contributed to the death of a patient. The Article does not provide an exact definition of "unusual death" [3].

Despite lack of a standardized definition of unusual death, physicians currently are required under Article 21 to report unusual deaths that occur during the course of medical care to the police. Next-of-kin or others such as media personnel may also report such deaths to the police. After receiving a report, police open a criminal investigation and healthcare providers are typically charged with "professional negligence resulting in death" (Penal Code Article 211, Clause 1). Thereafter, the case is treated as any crime in Japan as described above.

Since the 1999 Hiroo case, the Japanese mass media has followed numerous cases of medical error. Physicians and other healthcare providers are often reminded of the reporting requirement. The public has continued to show interest in medical error. However, subsequent trends in reporting and prosecution have not been well described. The aim of this study was to describe 1) the trend in reporting medical error to the police by physicians, next-of-kin, and others in cases of patient death and 2) the trend in prosecution of healthcare providers for medical error during the period from 1998 to 2008 in Japan.

## Methods

We collected and analyzed data regarding the number of reports to the police of patient death and resulting prosecutions between 1998 and 2008 using data from the National Police Agency database [4]. We grouped the reports into those made by physicians, next-of-kin, or others (newspaper reporters, etc.). From the same database, we also gathered information regarding the number of reported cases that were forwarded for prosecution. Because the statute of limitations for prosecution is 5 years from time of report, the number of prosecutions in a given year does not necessarily relate directly to the number of reports in the same year. These data are the only publicly available information source regarding the number of reports and prosecutions.

Statistical analyses were completed using SPSS version 16.0 (SPSS, Inc., Chicago IL). Because the aim of our research was to determine the trend of reporting and prosecution rates over time, annual linear regression models were used. The linear regression slope represented the annual change in reporting and prosecution, and the associated P value tested the null hypothesis that the slope was zero (no trend).

## Results

### Number of patient deaths reported to the police

**Figure 1:**
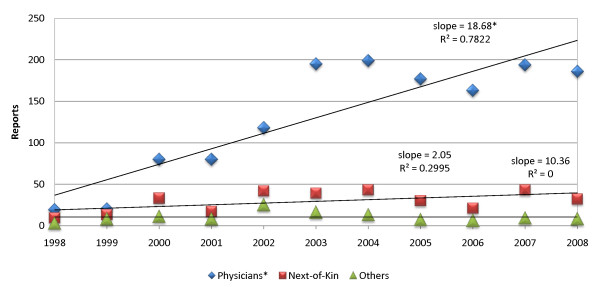
**Annual reports of patient deaths to the police**. Only reports of patient deaths made by physicians increased significantly from 1998 to 2008. The reports by next-of-kin and others such as media personnel remained at a low, constant rate. * Statistically significant increase, P < 0.001.

Figure 1 depicts reports of medical error to the police over the period from the beginning of 1998 to the end of 2008. The number of reports by physicians increased significantly (slope 18.68, R^2 ^= 0.78, P < 0.001). There was no significant increase in reports made by next-of-kin or others over the study period. Mean annual reports by group were as follows: Physicians 130.1 (± 70.1), next-of-kin 29.3 (± 12.5), and others 10.4 (± 6.0).

### Number of prosecutions

**Figure 2:**
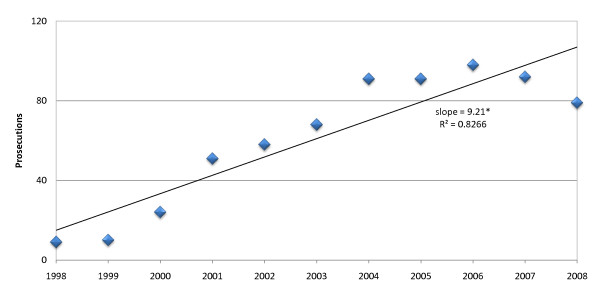
**Annual prosecutions of healthcare providers**. Prosecutions of healthcare providers increased significantly from 1998 to 2008. * Statistically significant increase, P < 0.001.

Figure 2 depicts the total number of resulting prosecutions of healthcare providers for medical error leading to patient death over the period from the beginning of 1998 to the end of 2008. The number of prosecutions increased significantly (slope 9.21, R^2 ^= 0.83, P < 0.001) over the study period. The mean annual prosecution rate was 61.0 (± 33.6).

## Discussion

From 1998 to 2008, patient deaths reported to the police by physicians increased to a statistically significant extent. The number of prosecutions also increased to a statistically significant extent. While the primary aim of our study was to determine if reporting and prosecutions have increased over the study period, careful inspection of the time series shows a rapid increase in both reporting and prosecution rates beginning in 2000 and extending through 2004, followed by a leveling off at what appears to be a new steady state. To our knowledge, there were no particular events around 2004 that would explain these results; perhaps the police, prosecutors, and courts reached their case load limits. Nonetheless, we have demonstrated that physicians are reporting to the police more and healthcare professionals are being prosecuted more.

The reason physicians are reporting more is likely multi-factorial. The 21st Article of the medical practitioner's law requires physicians to report to the police if there is an "unnatural death". Because the media has widely covered the Hiroo case and other similar cases, physicians have become familiar with this legal reporting requirement. Fear of prosecution under this law is often cited as the reason for the increase in physician reporting. Undoubtedly fear motivates some physicians to report to the police. But it is also possible that other reasons contribute. Physicians could simply desire to be "good citizens" and comply with the law, therefore reporting more often. Bereaved family members may pressure physicians by threatening to go to the police if physicians do not report. Hospital administrators may also have protocols in place that require physicians to report as a safeguard to avoid media attention and a tarnished reputation should non-compliance with the law be made public.

Unlike physicians, next-of-kin and others are not reporting more. It has been generally assumed that reporting by patient next-of-kin has increased following widespread media coverage that spurs public outrage surrounding medical error [5], and that this in turn has fueled physician prosecutions. Granted, people face significant barriers to pursuing civil litigation in Japan, such as high start-up costs, lengthy trials, and low chance of success [6]. In this light it seems plausible that, left with no other avenue of recourse, bereaved family members may be forced to pursue criminal litigation. However, our research does not support this assumption as the number of reports made directly by next-of-kin has not increased to a statistically significant degree since 1998. Of course, it is possible that family members indirectly cause physicians to report more by threatening to go to police themselves.

Because reporting by physicians has increased while all other reporting has remained constant, the increase in physician reporting likely fuels the increase in prosecutions. However, two things make the true cause difficult to decipher. First, the reports in a given year and the prosecutions in a given year are not necessarily directly related given that the statute of limitations allows for prosecution up to five years following report. Second, it is unknown what fraction of prosecutions results from physician reports vs. next-of-kin or other reports and therefore if the source of the report is associated with the likelihood of prosecution. Nevertheless, because the total number of prosecutions is greater than the number of reports by next-of-kin or others combined, it is clear that the physician reporting has played a large part in the rise in prosecutions. Even if one were to assume that 100% of reports made by next-of-kin and others resulted in prosecution, this could not account for the increase seen in prosecutions.

This rise in prosecutions is significant because a large number of prosecutions have resulted in criminal trial and conviction. According to data from the Public Prosecutors Office [7], 23% of the cases sent for prosecution of medical error eventually resulted in criminal trial (5% full trials, 18% summary trials). Whenever a case goes to trial, it is likely to end in conviction as the conviction rate in Japan for any criminal offense is known to be high compared to the rest of the world. Reasons for this may include public prosecutors only pursuing cases that are sure to bring conviction, acquittal having a negative impact on the careers of judges, and Japan lacking an impartial jury system [8]. Conviction rates specifically in cases of medical error are 90% or above (physicians, 93%, nurse 90%) [9]. Given these data, one can predict that of the roughly 100 cases sent for prosecution per year, 23 will lead to trial and 21 to conviction.

Japan is not only unique in its high conviction rates but also that gross negligence or deviation from standards of care have not been consistent elements in prosecuted cases. To be sure, prosecution of medical error occurs in other countries [10]. Between 1975 and 2005 in the United Kingdom, 44 physicians have been criminally prosecuted and 14 were convicted. Roughly 25 cases from 1982 to 2001 in the United States were prosecuted [11]. However, these cases typically have involved extreme negligence, such as an intoxicated surgeon killing a patient [12]. In contrast, the high profile 2004 Ono Hospital case is one example of many sent for prosecution despite no evidence of negligence. In the case, a patient died following caesarean-section complicated by hemorrhage related to placenta accreta. The physician involved was arrested and prosecuted for the patient death because he did not immediately perform a hysterectomy. He was later acquitted after judges determined he had acted well within accepted standards of care - many factors affect whether hysterectomy would be the most appropriate treatment course [13].

Because of this and other similar cases, medical providers are adopting defensive medical practices [14]. Physicians are turning away high-risk patients from emergency departments [15]. In thousands of cases per year, patients in ambulance transport are turned away from more than 11 hospitals before being accepted. The most frequent reason cited for refusal is not lack of physicians but "difficulty of treatment" according to data published by the Fire and Disaster Management Agency [16]. Pointing to the inherent unfairness of being prosecuted for providing routine medical care having undesirable outcomes, physicians are lobbying the government to intervene.

The Ministry of Health, Labor, and Welfare responded in 2008 by introducing a proposal for a new "third party" system that would replace the Article 21 requirement to report patient death to police [17]. In place of the current system, physicians would be required to report to an official arbitration body called the "Medical Safety Investigation Committee," composed of pathologists, internists, lawyers, patient advocates, etc. Following autopsy and investigation, the committee would make an official report with the aim of reducing medical error in the future. The committee would also forward the cases with elements of gross negligence or deviation from standards of care to the police for criminal prosecution. Whether this plan will be adopted or not remains to be seen. On separate fronts, organizations like the Japan Council for Quality Health Care are requiring accredited hospitals, including all government supported hospitals, to anonymously report physician errors leading to patient harm, death or otherwise, in order to improve patient safety [18].

Our research clarifies the trends in reporting and prosecution rates but does not provide any concrete information about why this change has occurred, the risk factors associated with reporting or prosecution, or whether this change is beneficial or harmful to patients or physicians. Future research should clarify these points.

## Conclusions

Reports of patient death to the police by physicians have significantly increased during the period from 1998 to 2008. Reports made by family members and others have not significantly increased. Resulting criminal prosecution of healthcare providers for medical error leading to patient death has also significantly increased from 1998 to 2008. The reasons for these increases are not well understood and should be investigated further.

## Competing interests

The authors declare that they have no competing interests.

## Authors' contributions

MS completed data acquisition. JS completed statistical analyses. JS and MS both contributed to the study conception and design, drafted the manuscript, and read and approved the final manuscript.

## Pre-publication history

The pre-publication history for this paper can be accessed here:

http://www.biomedcentral.com/1472-6963/10/53/prepub
